# Solubility and Leaching Risks of Organic Carbon in Paddy Soils as Affected by Irrigation Managements

**DOI:** 10.1155/2013/546750

**Published:** 2013-07-01

**Authors:** Junzeng Xu, Shihong Yang, Shizhang Peng, Qi Wei, Xiaoli Gao

**Affiliations:** ^1^State Key Laboratory of Hydrology-Water Resources and Hydraulic Engineering, Hohai University, Nanjing 210098, China; ^2^College of Water Conservancy and Hydropower Engineering, Hohai University, Nanjing 210098, China

## Abstract

Influence of nonflooding controlled irrigation (NFI) on solubility and leaching risk of soil organic carbon (SOC) were investigated. Compared with flooding irrigation (FI) paddies, soil water extractable organic carbon (WEOC) and dissolved organic carbon (DOC) in NFI paddies increased in surface soil but decreased in deep soil. The DOC leaching loss in NFI field was 63.3 kg C ha^−1^, reduced by 46.4% than in the FI fields. It indicated that multi-wet-dry cycles in NFI paddies enhanced the decomposition of SOC in surface soils, and less carbon moved downward to deep soils due to less percolation. That also led to lower SOC in surface soils in NFI paddies than in FI paddies, which implied that more carbon was released into the atmosphere from the surface soil in NFI paddies. Change of solubility of SOC in NFI paddies might lead to potential change in soil fertility and sustainability, greenhouse gas emission, and bioavailability of trace metals or organic pollutants.

## 1. Introduction 

Dissolved organic carbon (DOC) in soil, which is present in soil solution and interacts with colloids and clays, plays an important role in soil carbon cycling [1, 2]. It is highly related to the greenhouse gas (CO_2_ and CH_4_) emissions [3–5], nutrient availability [6, 7], as well as the mobilization, translocation, and toxicity of several inorganic and organic pollutants in soil [8–13]. The DOC losses from soil ecosystems, via leaching or surface runoff, account for numerous pollution problems of surface water and groundwater [14, 15]. Agricultural practices impact the timing and magnitude of DOC export from soils to rivers or ditches [16–18]. However, information on the effects of agricultural practices on soil DOC leaching is still limited, although it is a crucial component of the ecosystem carbon balance [19–22].

Rice is one of the most important crops in the Asian monsoon region [23]. The rice field ecosystem is commonly characterized by flooding conditions and high percolation rate. A great deal attention is paid to nitrogen and phosphorus with regard to leaching risks of nutrients in rice fields [24–28]. Leaching loss of DOC from paddy soil, which is relatively rich in organic matter, is always overlooked. With increasing water scarcity, water saving irrigation techniques, such as nonflooding controlled irrigation (NFI), alternate dry-wet irrigation (AWDI), and the rice intensification (SRI) system, are applied widely [29–33]. Soil wetting and drying cycles influence a large number of biological and chemical processes [34–37]. Solubility of soil organic carbon (SOC) and its leaching risks will change when the rice field is exposed to nonflooding conditions under water-saving irrigation management.

In the present study, water extractable organic carbon (WEOC) contents in soils and DOC in soil solutions, as well as DOC leaching risks, were measured in rice paddies under different irrigation managements to reveal the influence of NFI on soil DOC dynamics.

## 2. Materials and Methods

### 2.1. Site Description and Experimental Design

The study was conducted in rice paddies at the Kunshan irrigation and drainage experiment station (31°15′15′′N 120°57′43′′E) in the Tai Lake region in China. The study area has a subtropical monsoon climate, with an average annual air temperature of 15.5°C and a mean annual precipitation of 1,097.1 mm. The paddy soil is Gleyic-Stagnic Anthrosols, developed from alluvial deposits. The soil texture in the plowed layer (0–20 cm) is clay, with a total nitrogen content of 1.03 g kg^−1^, total phosphorus content of 1.35 g kg^−1^, total potassium content of 20.8 g kg^−1^, and pH of 7.4 (soil : water = 1 : 2.5 by weight). SOC contents for soil depths of 0–10, 10–20, 20–40, and 40–60 cm are 13.8, 12.1, 11.4, and 10.3 g kg^−1^; soil bulk densities are 1.28 g, 1.33^3^, 1.36, and 1.35 g cm^−3^, respectively. The saturated soil water contents (v/v) for the layers of 0–20, 0–30, and 0–40 cm are 52.4, 49.7, and 47.8%, respectively. The cropping system used is a rice-wheat rotation system. Winter wheat was harvested on 16-17 May before the experiment. The wheat straw was removed, whereas the root and about 10 cm stubble were buried by plowing. The variety of rice planted was Japonica Rice NJ46. The rice was transplanted with 13 cm × 25 cm hill spacing on 23 June, and harvested on 26 October in 2009.

Two irrigation treatments were used, namely, flooding irrigation (FI) and nonflooding controlled irrigation (NFI). A randomized complete block design and three replications were established in 6 plots (5 m × 7 m). The adjacent plots were separated by plastic membrane which was inserted into the ridges at a depth of 500 mm, to isolate the water within different plots and avoid hydraulic exchange between adjacent plots. In the FI rice fields, a depth of 3–5 cm standing water was always maintained after transplanting, except when drying in the later tillering and yellow maturity periods. In the NFI rice fields, standing water depth was kept between 5 and 25 mm during the first 7-8 days after transplanting (DAT) in regreening period; irrigation was applied only to keep soil saturated in other stages. Standing water was avoided in other stages, except during rain harvesting period and the pesticide or fertilizer application period.  Table 1 presents the root zone soil water content criteria in different growth stages. The same fertilizer doses for each split were applied into each plot according to the local conventional fertilizer application method.

### 2.2. Field Measurements

Irrigation water volumes were recorded by water meters installed on the pipes. Soil moisture in rice field was monitored with three replications using a time domain reflectometer (TDR, soil moisture, USA) and with 20 cm waveguides installed at 0–20, 20–40, and 40–60 cm depths. Water layer depth was monitored using a vertical ruler fixed in the field. Daily meteorological data, including precipitation volume, wind speed, temperature (maximum, minimum, and average), sunshine duration, and relative humidity, were recorded by an automatic weather station (ICT, Australia). Soil temperature and soil redox potentials (Eh) at 5 cm depth were measured using mercury thermometers and oxidation-reduction potential meters, with three replications in situ. Rice was harvested on 26 October 2009, and yield was determined for each plot. 

### 2.3. Soil Sampling and WEOC Contents Measurement

Soil samples were collected during the rice season with a hand auger for soil WEOC measurement. The sampling was conducted at five locations for each treatment plot at five depths, 0–10, 10–20, 20–30, 30–40, and 40–60 cm. Then fresh samples of the same depth in each plot were homogenized by mixing and separated from debris and crop residues. Five grams of fresh soil samples were then extracted in distilled water (soil : water = 1 : 10 by weight) on a shaker for 60 min. DOC in the extract was determined using a TOC-1020 A analyzer (Elementar, High TOC II, Germany). Using the same method, soil samples were collected at pretransplanting and postharvesting periods in depths of 0–10, 10–20, 20–40, and 40–60 cm for SOC measurement. Ten grams of fresh soil samples were selected for SOC measurement using the potassium dichromate oxidation method, with 0.8 mol L^−1^ K_2_Cr_2_O_4_-H_2_SO_4_ solution at 170−180°C (oil bath) [38]. Soil samples moisture contents were determined using an oven-dried method, and WEOC and SOC contents were calculated as milligram C per gram dry soil. Change in SOC storage was calculated based on the values obtained at pretransplant and at harvest.

### 2.4. Soil Solution Sampling and DOC Contents Measurement

Ceramic suction cups (2 cm in inner diameter and 7 cm in length) with numerous pores (about 2 *μ*m in diameter) were installed vertically at 7–14 cm, 27–34 cm, and 47–54 cm depths to collect soil solutions with three replications. To acquire a field-equilibrated status and eliminate the sorb of DOC by suction cups [39], the cups, which were cleaned by 0.1 molar HCl and deionised water, were installed firmly into the soil one year ago in June 2008 [28]. The clay suction cup was embedded in a polyvinyl chloride pipe, allowing the water to be pumped out. Soil solutions were collected and stored in 100 mL polytetrafluoroethylene bottles and then taken to the laboratory immediately. DOC contents in the soil solutions were determined using the TOC-1020 A analyzer. 

### 2.5. Leaching Loss of DOC

Seasonal DOC leaching losses were calculated based on DOC contents in 47–54 cm soil solutions and deep percolation (DP) rate. Daily DP was calculated by following the water balance principle based on field measurements:

(1) $$\begin{array}{c} {\text{DP}}_{t}=W_{t-1}-W_{t}+I_{t}+P_{t}-D_{t}-{\text{ET}}_{t}, \end{array}$$

where DP is the volume of the percolation water, *W* is the flooding depth or the soil water content in the root zone. *I*, *P*, and *D* are the water volumes of irrigation, precipitation, and drainage, respectively. ET is the evapotranspiration, which was calculated using the water balance principle based on measurement in bottom-sealed lysimeters (nonweighted, 40 cm in diameter and 60 cm in depth with 4 rice hills) with the same irrigation management as the plot.

## 3. Results

### 3.1. Water Regimes and Soil Characteristics

**Figure 1:**
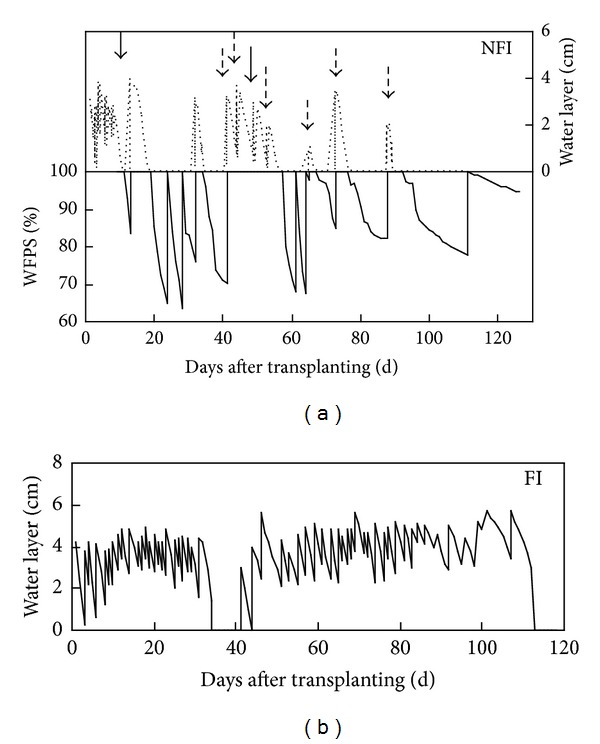
Water depth and soil moisture in FI and NFI rice fields (solid and dashed arrows denote irrigations for fertilizer and pesticides application in NFI rice fields).

**Figure 2:**
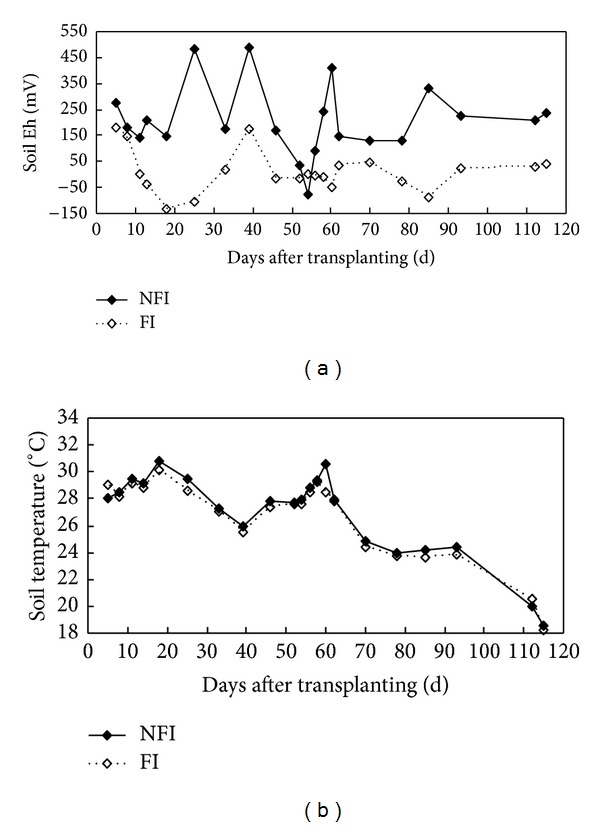
Seasonal variation of soil Eh and soil temperature at 5 cm depth in rice fields under different water managements.

Eleven wet-dry cycles were observed in NFI fields, with more than 72 days of nonflooding (Figure 1). About two-thirds of the total growth season was nonflooding in the NFI fields, which was much longer than those reported in zero-drainage or alternate wetting and drying irrigation rice fields [40, 41]. Multi-wet-dry cycles led to huge change in soil properties in the NFI fields. Soil redox potentials (Eh) at 5 cm depth ranged from −77.9 to +488.9 mV in the NFI fields, much higher than those in the FI fields (from −134.43 to +181.86 mV) (Figure 2). Drying in the NFI fields was always accompanied by a rapid increase in Eh values, whereas rewetting caused a sharp decrease. Soil Eh at 5 cm depth increased from a negative value to as high as +480 mv in the NFI fields (25 DAT and 39 DAT). In the FI paddies, midseason drainage also led to a significant Eh increase, from −104.1 to +131.1 mV (33–39 DAT). Soil temperatures at 5 cm depth were slightly higher in the NFI fields than those in the FI fields during most of the rice season (Figure 2). 

### 3.2. Rice Yields and Water Consumption

Evapotranspiration and percolation were 404.6 and 368.8 mm in the NFI fields, which were reduced by 111.7 and 276.8 mm compared with the FI treatment (Table 2). Irrigation volumes in the NFI and FI fields were 233.3 and 635.9 mm, whereas water consumption volumes were 773.4 and 1,161.9 mm, respectively. Irrigation volumes and water consumption in the NFI fields were reduced by 63.3 and 33.4%, compared with the FI fields. Rice yield for the NFI treatment was 10,335.8 kg ha^−1^; it was the same as the yield for FI treatment (9,889.7 kg ha^−1^). Water use efficiency greatly increased in the NFI paddies due to the large reduction in water consumption and irrigation volume. It indicated that NFI is able to get the same yield as FI with a lower cost irrigation and higher water use efficiency than FI.

### 3.3. Soil WEOC Contents

**Figure 3:**
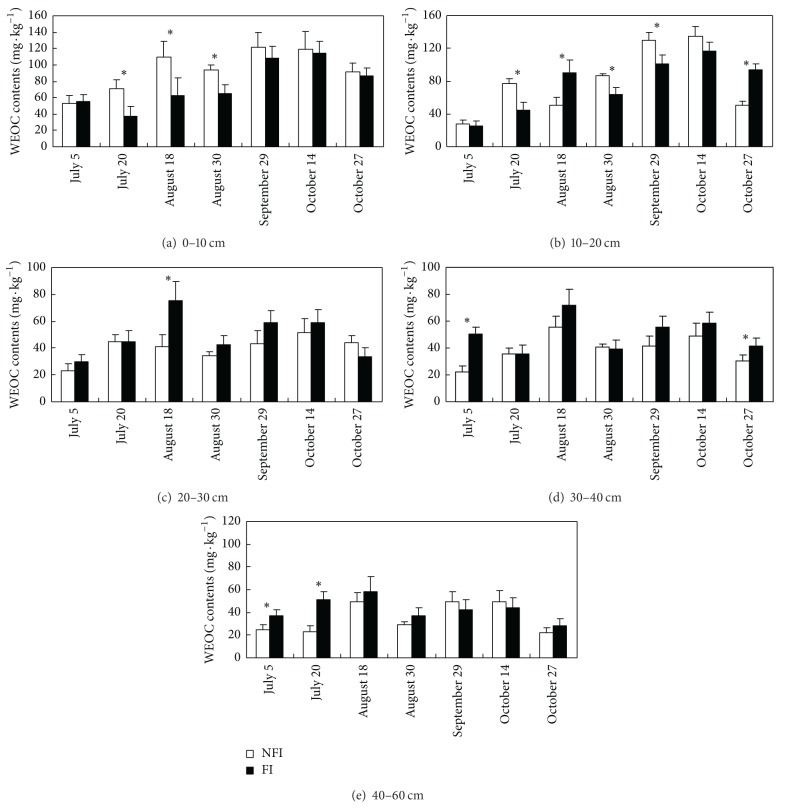
Water extractable organic carbon (WEOC) contents in paddy soils under different irrigation managements (*indicates difference between NFI and FI that is significant at *P* < 0.05).

Soil WEOC contents in both FI and NFI fields at different stages decreased with the increase of soil depth (Figure 3). Soil WEOC contents were more variable in the top 0–20 cm soils than in the 30–40 cm and 40–60 cm soils. Soil WEOC contents were always high in the middle stage, when the crop growth and agronomic activities were intensive. Soil WEOC contents at 0–10, 10–20, 20–30, 30–40, and 40–60 cm depths varied in the range of 52.4–121.0, 27.4–134.5, 23.5–51.3, 22.2–55.8, and 22.2–49.7 mg C kg^−1^ in the NFI fields, whereas those in the FI fields varied in the range of 37.7–114.0, 25.0–116.5, 29.6–75.6, 35.3–71.7, and 28.4–58.0 mg C kg^−1^. But the WEOC contents in the NFI and FI fields were lower than the results obtained by Zhan et al. (2010) [42] in paddy soils (0.44–0.83 g C kg^−1^) in Hubei China. 

WEOC contents in surface soils at 0–10 and 10–20 cm depths in the NFI fields were mostly significantly higher than those in the FI fields. However, WEOC contents in the NFI fields were frequently lower than in FI fields in deep soils at 20–30, 30–40, and 40–60 cm depths, but only a few results are significantly lower. Soil WEOC contents at 0–10 cm and 10–20 cm depths in the NFI fields increased by an average of 18.5 mg C kg^−1^ (24%) and 2.7 mg C kg^−1^ (4%) compared with those in the FI fields. Soil WEOC contents at 20–30, 30–40, and 40–60 cm depths in the NFI fields decreased by 8.7 mg C kg^−1^ (18%), 11.0 mg C kg^−1^ (22%), and 7.3 mg C kg^−1^ (17%). Therefore, the long duration of nonflooding aerobic condition and multi wet-dry cycles in NFI enhanced the soil organic decomposition and mineralization at 0–20 cm depth.

### 3.4. DOC Concentrations in Soil Solutions

**Figure 4:**
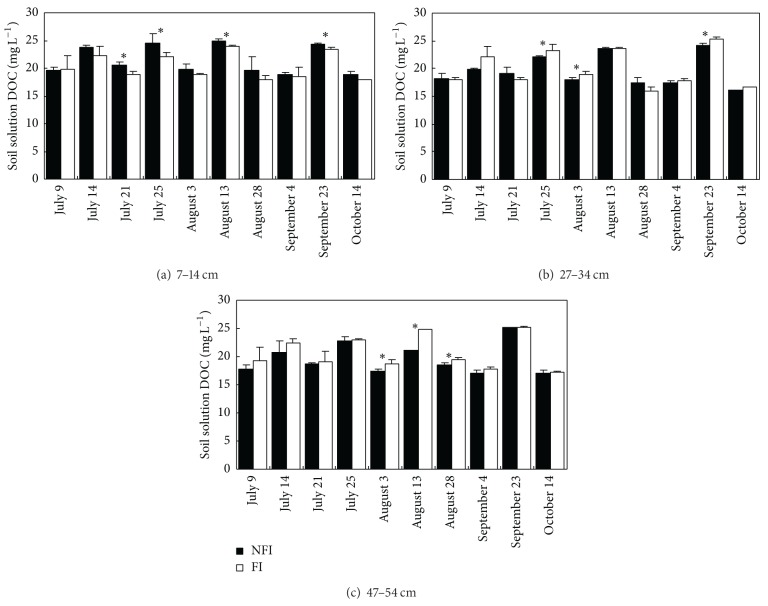
Dissolved organic carbon (DOC) concentrations in paddy soil solutions at different depths under different irrigation managements (*indicates difference between NFI and FI that is significant at *P* < 0.05).

DOC concentrations of soil solutions were slightly higher in surface soils than in deep soils (Figure 4). Compared with FI soils, DOC concentrations in soil solutions at 7–14 cm depth in NFI paddies were slightly higher, which increased by 0.81–2.49 mg L^−1^. The DOC concentrations in soil solutions further confirm that long duration of nonflooding aerobic condition and wet-dry cycles in NFI enhanced the soil organic decomposition and mineralization in surface soils. DOC concentrations in NFI soil solutions at 47–54 cm depth decreased slightly by 0.05–3.61 mg L^−1^ compared with those in FI paddies, because the decreased percolation led to less carbon moving downward to deep soils. However, the DOC concentrations in NFI soil solutions at 27–34 cm depth were highly variable, sometimes higher than those in FI and sometimes lower. But generally there is no significant difference between DOC concentrations in NFI and FI fields, with only a few number of differences are significant between the two treatments.

### 3.5. Leaching Risks of DOC

**Figure 5:**
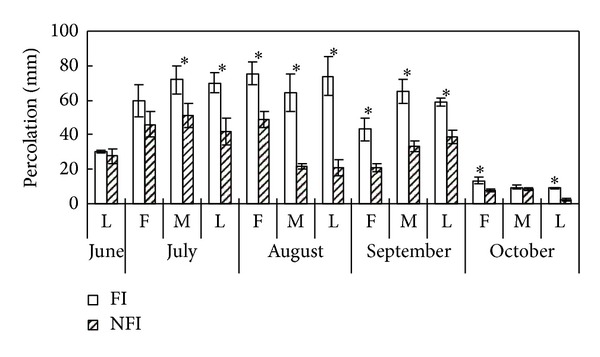
Percolation volumes from paddy soils under different irrigation managements (*indicates difference between NFI and FI that is significant at *P* < 0.05).

Ten-day deep seepage ranged from 2.3 mm to 46.2 mm in NFI fields, which was significantly lower than the corresponding values (from 7.2 mm to 72.6 mm) in FI fields except in later June and middle October (Figure 5). Seasonal percolation in NFI paddies was 368.8 mm, which was reduced by 42.9% compared with the FI treatment. Seasonal leaching loss of DOC was 63.3 kg C ha^−1^ from NFI soils, which was reduced by 46.4% compared with those from FI fields (118.1 kg C ha^−1^). Although DOC concentration in surface soil (0–20 cm) and soil solutions (7–14 cm) was increased in NFI fields, the reduced percolation in the NFI fields led to lower risk of DOC leaching loss than in FI fields. Several studies focused on DOC losses from forest soil [21, 43]. However, few studies on DOC leaching losses in rice paddies have been reported. In the current study, the seasonal leaching losses of DOC from FI paddies fell in the range reported by Katoh et al. (2004) [44] in typical rice fields in Japan (85 to 170 kg C ha^−1^). The seasonal DOC leaching losses of FI paddies were less than the lower limit of 85 kg C ha^−1^.

### 3.6. Soil Organic Carbon

**Figure 6:**
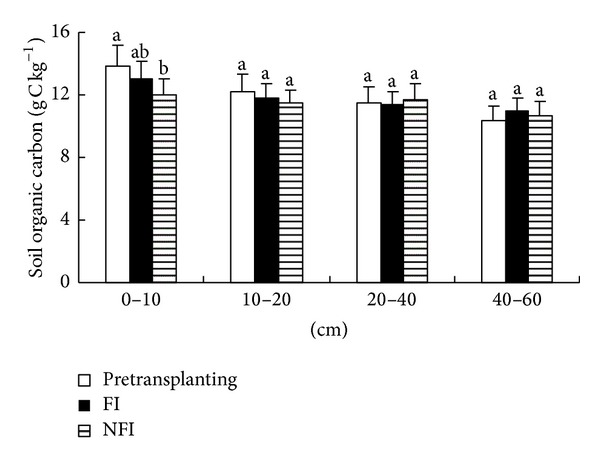
Soil organic carbon (SOC) contents in paddy soils under different irrigation management (pretransplanting means the SOC contents in soil before transplanting. Difference between the columns with the same letter in the same soil depth is not significant at *P* < 0.05).

SOC contents at harvest were reduced at 0–10 and 10–20 cm depth for both treatments but increased at 20–40 and 40–60 cm depth in the NFI and FI fields, respectively (Figure 6), but the reduction is insignificant in the short-term experiment except for 0–10 cm soil in NFI paddies. The SOC in different soil depth was calculated based on the SOC content (in Figure 6) and soil bulk density. The SOC storage in NFI paddies was reduced by 231 and 96 g m^−2^ at 0–10 cm and 10–20 cm depths but increased by 59 and 83 g m^−2^ at 20–40 and 40–60 cm depths. SOC storage in FI paddies was reduced by 118, 66, and 52 g m^−2^ at 0–10, 10–20, and 20–40 cm, and increased by 161 g m^−2^ at 40–60 cm. The obtained values confirm that long duration of nonflooding aerobic condition and wet-dry cycles enhanced the soil organic decomposition and mineralization in paddy soils, leading to increased SOC loss [45, 46]. Compared with FI paddies, less carbon was accumulated in deep soils (40–60 cm), and more SOC was lost in surface soils (0–20 cm) of NFI paddies. SOC content of NFI paddies indicate that more carbon was released into the atmosphere from surface soil than in FI paddies. A study regarding the greenhouse gas emission from NFI paddies [47] reported that seasonal CH_4_ emission from NFI paddies was 1.17–1.35 g m^−2^, which was much lower than that (6.62–7.20 g m^−2^) from FI paddies. Thus, we can deduce that more CO_2_ was released from NFI paddies than FI paddies because the aerobic condition favored carbon decomposition [45]. 

## 4. Discussions

### 4.1. Solubility and Mobility of Soil Organic Carbon

The long duration of nonflooding aerobic condition and multi-wet-dry cycles in NFI enhanced the solubility of organic carbon in 0–20 cm soil (Figures 3 and 4). Several studies confirmed the effect of wet-dry cycles, oven drying, or air drying with incubation experiments by measuring the soil carbon mineralization rate, soil respiration, soil microbial biomass carbon [35, 37, 48–51], or soil DOC contents [52–54]. Higher WEOC contents in deep soils of 20–30, 30–40, and 40–60 cm in the FI fields indicate that more DOC was transferred from the topsoils to the deep soils due to high percolation rate (Table 2). The relationship between soil WEOC distribution in the soil profile and water flows was indicated by Mertens et al. (2007) [55] and Junod et al. (2009) [56] on arable soils. But DOC contents in deep soil solutions in NFI paddies were always lower than those in FI paddies (Figure 4). It indicated that downward moving of DOC was determined more by deep seepage volume than the DOC contents in surface soils. 

### 4.2. Potential Environment Impacts

High WEOC and DOC contents in NFI soils are the consequence of the high microbial oxidative breakdown of soil organic matter and turnover of microbial biomass [2]. The high microbial activity in NFI soil will be accompanied with high soil respiration [57, 58], which led to greenhouse gas (CO_2_ and CH_4_) emission. The lower SOC contents in NFI surface soil also confirmed it. Compared with FI paddies, less carbon was accumulated in deep soils (40–60 cm) and more SOC was lost in surface soils (0–20 cm) of NFI paddies (Figure 6), although the differences were mostly insignificant for one-year experiment in current research. If the NFI is applied to rice paddies in long-term, the effect on soil carbon pool and soil carbon output will be accumulated and get even significant. The reduced SOC content in NFI surface soils indicates that more carbon was released into the atmosphere from surface soil than in FI paddies. A study regarding the greenhouse gas emission from NFI paddies [47] reported that seasonal CH_4_ emission from NFI paddies was 1.17–1.35 g m^−2^, which was much lower than that (6.62–7.20 g m^−2^) from FI paddies. Thus, we can deduce that more CO_2_ was released from NFI paddies than FI paddies because the aerobic condition favored carbon decomposition [45]. The reduced percolation in the NFI fields also led to lower DOC leaching loss than in FI fields that is helpful to reduce the risk of groundwater pollution. In addition, solubility of SOC concentration (especially DOC content) is also an important factor for the translocation of trace metals [11–13] and organic compound pollutants [8–10]. Thus, soil respiration rate, SOC fractions, and translocation of heavy metals and organic compounds should be studied to help illustrate the ecoenvironment effect of water saving irrigation on rice paddies.

### 4.3. Soil Fertility and Sustainability

Generally, flooding condition in rice paddies frequently results in high SOC contents compared with the upland's seasonal soil carbon accumulation, or results in long-term SOC continuous accumulation [59–61]. As a result of enhanced decomposition and mineralization of SOC in NFI surface soil, SOC in surface NFI soil decreased. Long-term application of NFI in rice fields might lead to more release of carbon from surface soil and consequently lead to degradation in the soil fertility and sustainability. Thus, future studies should look into the combinations of water and carbon (residue or biochar) management practices to enhance soil carbon storage and soil sustainability in NFI rice paddies.

## 5. Conclusions

WEOC contents in soils and soil solutions, soil organic carbons, and DOC leaching risks were observed in rice paddies under different irrigation managements. The results indicated that long duration of nonflooding aerobic condition and wet-dry cycles in NFI enhanced the soil organic decomposition and mineralization and consequently led to high solubility of SOC in surface soil. WEOC contents in soils and DOC in soil solution increased in NFI paddies in the surface soil layer but decreased in the deep soil layer. Less carbon moved downward to deep soils due to the decrease in percolation. The leaching losses of DOC in NFI fields were reduced by 46.4% compared with those from FI fields. SOC in surface soil was decreased in NFI paddies, indicating that more carbon was released into the atmosphere from the surface soil than in FI. The influence of irrigation management on soil organic carbon dynamics, soil respiration, and net CO_2_ exchange are important problems that should be discussed in future studies. Moreover, the influence on soil carbon fraction, which strongly related to changes in translocation of heavy metals and organic compounds, must also be considered.

## Figures and Tables

**Table 1:** Limits for irrigation in different stages of rice for non-flooding controlled irrigation.

Stages	Regreening	Tillering			Jointing and booting		Heading and flowering	Milk maturity	Yellow maturity
		Former	Middle	Later	Former	Later			
Upper limit	30 mm	*θs*_1_	*θs*_1_	*θs*_1_	*θs*_2_	*θs*_2_	*θs*_3_	*θs*_3_	Drying
Lower limit	10 mm	0.7*θs*_1_	0.65*θs*_1_	0.6*θs*_1_	0.7*θs*_2_	0.75*θs*_2_	0.8*θs*_3_	0.7*θs*_3_	
Monitored soil depth (cm)	—	0–20	0–20	0–20	0–30	0–30	0–40	0–40	—

*θs*_1_, *θs*_2_, and *θs*_3_ are the saturated water content of the soil in different stages of rice.

**Table 2:** Rice yields and water consumption under different irrigation managements.

Treatment	Yield	Irrigation	Evapotranspiration	Water consumption	Deep seepage
	Kg ha^−1^	mm	mm	mm	mm
NFI	10335.8^a^	233.3^a^	404.6^a^	773.4^a^	368.8^a^
FI	9889.7^a^	635.9^b^	516.3^b^	1161.9^b^	634.7^b^

Different letters in each column represent significant difference between the treatments at *P* = 0.05 by *t*-test.
